# Establishment of a TGFβ-Induced Post-Transcriptional EMT Gene Signature

**DOI:** 10.1371/journal.pone.0052624

**Published:** 2012-12-20

**Authors:** George S. Hussey, Laura A. Link, Andrew S. Brown, Breege V. Howley, Arindam Chaudhury, Philip H. Howe

**Affiliations:** 1 Department of Biochemistry and Molecular Biology, Medical University of South Carolina, Charleston, South Carolina, United States of America; 2 Hollings Cancer Center, Medical University of South Carolina, Charleston, South Carolina, United States of America; 3 Department of Biological, Geological and Environmental Sciences, Cleveland State University, Cleveland, Ohio, United States of America; 4 Department of Biomedical Sciences, Kent State University, Kent, Ohio, United States of America; 5 Department of Molecular Physiology and Biophysics, Baylor College of Medicine, Houston, Texas, United States of America; University of Edinburgh, United Kingdom

## Abstract

A major challenge in the clinical management of human cancers is to accurately stratify patients according to risk and likelihood of a favorable response. Stratification is confounded by significant phenotypic heterogeneity in some tumor types, often without obvious criteria for subdivision. Despite intensive transcriptional array analyses, the identity and validation of cancer specific ‘signature genes’ remains elusive, partially because the transcriptome does not mirror the proteome. The simplification associated with transcriptomic profiling does not take into consideration changes in the relative expression among transcripts that arise due to post-transcriptional regulatory events. We have previously shown that TGFβ post-transcriptionally regulates epithelial-mesenchymal transition (EMT) by causing increased expression of two transcripts, *Dab2* and *ILEI*, by modulating hnRNP E1 phosphorylation. Using a genome-wide combinatorial approach involving expression profiling and RIP-Chip analysis, we have identified a cohort of translationally regulated mRNAs that are induced during TGFβ-mediated EMT. Coordinated translational regulation by hnRNP E1 constitutes a post-transcriptional regulon inhibiting the expression of related EMT-facilitating genes, thus enabling the cell to rapidly and coordinately regulate multiple EMT-facilitating genes.

## Introduction

Traditional gain-of-function and loss-of-function approaches have yielded an enormous amount of information in regards to gene function in mammalian development and disease. However, changes in mRNA levels are not always correlative with changes in protein abundance, underlying the importance of post-transcriptional regulation during control of gene expression and activity [1]. Indeed, during germ cell development, it has been demonstrated that the 3′-untranslated regions (3′-UTR), when fused to a reporter, are sufficient to confer temporo-spatial specificity for 80% of genes tested [2]. Thus, it is clear that the UTRs of mRNA transcripts can significantly impact gene expression. The ‘human genome project’ reported the mean lengths of 5′-untranslated regions (5′-UTRs) and 3′-UTRs of human mRNAs as 300nt and 770nt, respectively, compared to the mean coding length of 1340 nt [3], [4], generating renewed interest in the 3′-UTRs of mRNAs to map post-transcriptional regulatory activities.

The epithelial-mesenchymal transition (EMT), in which cells undergo a developmental switch from a polarized, epithelial phenotype to a highly motile fibroblastic or mesenchymal phenotype, has emerged not only as a fundamental process during normal embryonic development and in adult tissue homeostasis, but is also aberrantly activated during metastatic progression [5], [6], [7]. EMT is associated with changes in cell-cell adhesion, remodeling of extracellular matrix, and enhanced migratory activity, all properties that enable tumor cells to metastasize [5], [6], [7]. Numerous cytokines and autocrine growth factors, including TGFβ, have been implicated in EMT [8], [9]. Our previous studies [10], [11] and those of others [12], [13] have shown that regulation of gene expression at the post-transcriptional level plays an indispensable role during TGFβ-induced EMT and metastasis. We identified a transcript-selective translational regulatory pathway in which a ribonucleoprotein (mRNP) complex, consisting of heterogeneous nuclear ribonucleoprotein E1 (hnRNP E1) and eukaryotic elongation factor 1A1 (eEF1A1), binds to a 3′-UTR regulatory BAT (TGFβ activated translation) element and silences translation of *Dab2* and *ILEI* mRNAs, two transcripts which are involved in mediating EMT [10], [11]. TGFβ activates a kinase cascade terminating in the phosphorylation of hnRNP E1, by isoform-specific stimulation of protein kinase Bβ/Akt2, inducing the release of the mRNP complex from the 3′-UTR element, resulting in the reversal of translational silencing and increased expression of *Dab2* and *ILEI* transcripts.

We have previously shown that shRNA-mediated silencing of Dab2 and ILEI in normal murine mammary gland (NMuMG) cells is sufficient to inhibit TGFβ-mediated EMT as analyzed morphologically and by loss of upregulation of N-cadherin and vimentin, mesenchymal cell markers, whereas their overexpression does not induce constitutive EMT, independent of TGFβ signaling [10], [14]. Thus Dab2 and ILEI are required, but not sufficient, for TGFβ-induced EMT. Hence, we, and others based on our studies [15], hypothesized that there are other mRNAs that are being silenced by hnRNP E1 in a similar fashion, and which cumulatively contribute to TGFβ-induced EMT. To address this hypothesis, we adopted a combinatorial approach involving polysome profiling and RIP-Chip analyses using hnRNP E1 and filtered the array data based on the regulatory mechanism of Dab2 and ILEI. This led to the identification and validation of a cohort of target mRNAs that follow the same pattern of regulation as Dab2 and ILEI. Similar to Dab2 and ILEI, the identified target mRNAs harbor a structural BAT element in the 3′-UTR as revealed by *in silico* analysis. This cohort of mRNAs may represent a new TGFβ responsive and hnRNP E1-mediated regulon, operative at a post-transcriptional level in order to mediate TGFβ-induced EMT in a temporal and expedited fashion.

## Results

### Experimental Design and Identification of a TGFβ-induced Post-transcriptional EMT Gene Signature

**Figure 1:**
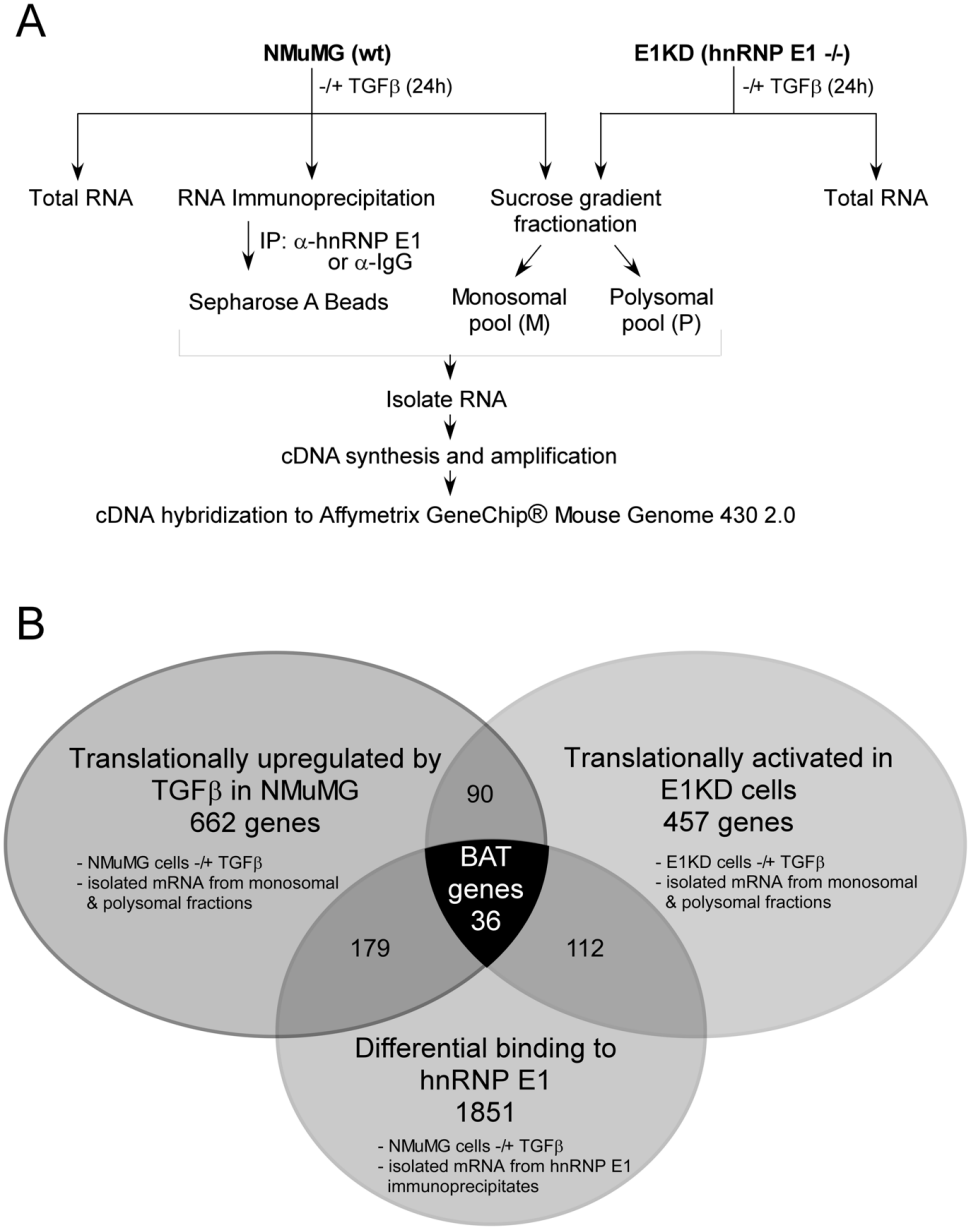
The experimental design involves a combinatorial approach using microarray analyses of polysome profiles and RNA Immunoprecipitation. (A) Flow chart representing the experimental design. For expression profiling, cytosolic extracts from untreated and TGFβ-treated (24 hr) NMuMG and E1KD cells were fractionated by sucrose gradient centrifugation and RNA was isolated from the non-translating monosomal pool and actively translating polysomal pool, designated as M and P, respectively (n = 2). Total unfractionated RNA was isolated from NMuMG and E1KD cells treated ± TGFβ (24 hr) (n = 2). For the RIP-Chip analysis, cytosolic extracts from untreated and TGFβ-treated (24 hr) NMuMG cells were immunoprecipitated with α-hnRNP E1 antibody or an isotype control (n = 2). (B) Venn diagram summarizing the results of the genome wide analysis. Intersection of the three data sets yielded 36 putative BAT genes whose expression is translationally regulated by TGFβ through hnRNP E1.

To identify potential target mRNA transcripts that are translationally regulated by hnRNP E1 in a TGFβ-dependent manner, we adopted a combinatorial approach involving expression profiling analyses and RNA immunoprecipitation analysis (RIP-Chip). As shown (Fig. 1A), we performed a screen using: 1) total mRNA and 2) RNA isolated from monosomal (non-translating) versus polysomal (translating) fractions from TGFβ-treated (24 h) and non-treated NMuMG cells and from the hnRNP E1 knockdown derivative (E1KD), that undergo constitutive EMT even in the absence of TGFβ [10], [11]. In addition, we screened for transcripts that selectively interact with hnRNP E1 in NMuMG cells under unstimulated conditions and subsequently lose their temporal association following TGFβ stimulation (Fig. 1A). The samples were individually hybridized to Affymetrix GeneChip® Mouse Genome 430 2.0 arrays.

Following normalization, data was filtered to produce three datasets representing 1) TGFβ translationally regulated genes, 2) genes translationally activated following hnRNP E1 knockdown and 3) hnRNP E1 interacting transcripts (Fig. 1B). Genes from the TGFβ translationally regulated dataset were selected as transcripts that displayed an enhanced ratio of association with the actively translating polysomal pool compared to the non-translating monosomal pool, with no or minor changes in total mRNA expression in NMuMG cells following TGFβ stimulation. mRNA transcripts that displayed enhanced association with polyribosomes irrespective of TGFβ-treatment in E1KD cells were candidates for translationally active genes in an hnRNP E1 knockdown context. Whereas, transcripts which displayed a decreased association with hnRNP E1 in NMuMG cells following TGFβ stimulation, as determined by RIP-Chip analysis, were selected as hnRNP E1 interacting candidates (Fig. 1B).

To perform a functional interpretation of our array analysis, all three datasets were queried against GO, Panther and KEGG databases using DAVID and Panther platforms (Table 1). Analysis of the TGFβ translationally regulated dataset revealed significant enrichment of categories associated with cell cycle, transcription and ubiquitin-mediated proteolysis. Genes actively translated in E1KD cells are involved in cell cycle, translation and the regulation of the actin cytoskeleton, whereas, transcripts that displayed differential interaction with hnRNP E1 mapped to terms associated with transcription, ubiquitin-mediated proteolysis, in addition to enrichment of several signaling pathways including MAPK, Wnt, integrin and Ras pathway. This analysis is consistent with our findings that TGFβ-mediated translational regulation plays a major role during EMT [10], [11], as evidenced by enrichment of EMT-associated processes and pathways. In addition, our data indicates that EMT-associated processes are coordinately regulated at both the transcriptional and post-transcriptional level. Enrichment of EMT-associated pathways within the E1KD and RIP datasets also suggest that hnRNP E1 is a key effector of TGFβ-mediated translational regulation.

**Table 1:** Number of genes from dataset assigned to a given biological process or pathway is compared to the number of genes expected by chance to map to the term.

Process/Pathway	Database	number of genes (expected number)	P-value
**TGFβ regulated dataset**			
Mitotic cell cycle	GO biological process	34 (8.3)	1.8 E-08
Cell division	GO biological process	36 (9.5)	4.6 E-08
Nucleoside, nucleotide and nucleic acid metabolism	Panther biological process	140 (87.5)	1.6 E-07
Cell cycle	GO biological process	54 (20.8)	5.0 E-07
Mitosis	Panther biological process	27 (9.0)	2.1 E-04
Transcription	GO biological process	101 (59.4)	2.4 E-04
RNA splicing	GO biological process	24 (6.9)	5.8 E-04
RNA processing	GO biological process	37 (14.8)	0.001
Cell cycle	KEGG pathway	16 (4.1)	0.002
mRNA metabolic process	GO biological process	29 (10.4)	0.003
Spliceosome	KEGG pathway	14 (4.0)	0.020
DNA metabolism	Panther biological process	21 (8.1)	0.030
Ubiquitin proteasome pathway	Panther pathway	8 (1.6)	0.043
Pre-mRNA processing	Panther biological process	20 (7.7)	0.046
**hnRNP E1 knockdown dataset**			
Nucleoside, nucleotide and nucleic acid metabolism	Panther biological process	87 (54.4)	8.4 E-04
Cell cycle	Panther biological process	33 (15.7)	0.014
Translation	GO biological process	20 (6.7)	0.046
Regulation of actin cytoskeleton	KEGG pathway	14 (4.5)	0.055
**hnRNP E1 RIP dataset**			
Regulation of transcription	GO biological process	268 (187.7)	1.6 E-10
Transcription	GO biological process	220 (137.5)	3.3 E-09
MAPK signaling pathway	KEGG pathway	43 (17.9)	2.6 E-05
Intracellular signaling cascade	Panther biological process	101 (59.4)	3.2 E-05
Regulation of RNA metabolic process	GO biological process	176 (117.3)	4.9 E-05
Intracellular protein traffic	Panther biological process	103 (64.4)	3.5 E-04
Ubiquitin mediated proteolysis	KEGG pathway	26 (9.3)	6.6 E-04
Wnt signaling pathway	Panther pathway	35 (14.6)	6.0 E-04
mRNA transcription	Panther biological process	178 (127.1)	0.001
Pathways in cancer	KEGG pathway	44 (22)	0.002
Colorectal cancer	KEGG pathway	19 (5.9)	0.002
Protein phosphorylation	Panther biological process	77 (48.1)	0.004
Nucleoside, nucleotide and nucleic acid metabolism	Panther biological process	276 (230)	0.004
B cell activation	Panther pathway	13 (3.3)	0.006
Protein catabolic process	GO biological process	76 (44.7)	0.008
EGF receptor signaling pathway	Panther pathway	17 (5.6)	0.013
Angiogenesis	Panther pathway	20 (7.5)	0.018
Ras Pathway	Panther pathway	12 (3.2)	0.022
Protein modification	Panther biological process	115 (82.1)	0.023
Regulation of Rho protein signal transduction	GO biological process	21 (6.8)	0.026
Focal adhesion	KEGG pathway	29 (13.8)	0.026
Endocytosis	Panther biological process	36 (18)	0.029
Integrin signalling pathway	Panther pathway	21 (8.4)	0.029
Natural killer cell mediated cytotoxicity	KEGG pathway	21 (8.4)	0.032
PDGF signaling pathway	Panther pathway	17 (6.3)	0.049

P-value adjusted for multiple testing using the Bonferroni method.

### Identification of Candidate mRNA Transcripts Translationally Regulated by hnRNP E1 in a TGFβ-dependent Manner

**Figure 2:**
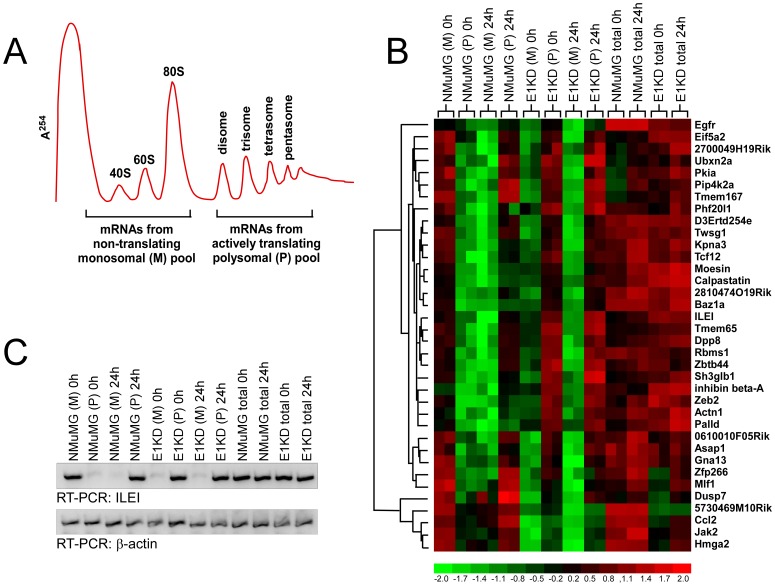
Quantitative data analysis demonstrates the translational status of the TGFβ-induced post-transcriptional EMT gene signature. (A) Schematic of polysome profile analysis. Monosomal fractions (M; 40S, 60S, and 80S fractions) and polysomal fractions (P) from NMuMG or E1KD cells treated ± TGFβ for 24 hr were isolated by sucrose gradient centrifugation and pooled. (B) Heatmap of the raw signal intensity values of the differential gene expression profile for the EMT signature genes compared to total, unfractionated mRNA. (C) RT-PCR analysis of microarray RNA samples was used to demonstrate the differential gene expression profile of ILEI.

In order to identify genes whose expression is translationally regulated by TGFβ through hnRNP E1, the intersection of our three data sets was utilized (Fig. 1*B*) revealing 36 genes, which we have termed BAT genes (Table 2). The translational status of the 36 putative BAT genes as determined by isolation of non-translating monosomal (M) fractions (40S, 60S and 80S) and actively translating polysomal (P) fractions from cells treated ± TGFβ for 24 h (Fig. 2A) is represented by the signal intensities of monosomal and polysomal association, and is displayed as a heat plot (Fig. 2B). The data reveal that the expression of these transcripts (total mRNA) did not vary significantly ± TGFβ in either the parental NMuMG or E1KD cells (Fig. 2B). However, in the NMuMG cells, these transcripts preferentially translocated from the M to P fractions following TGFβ stimulation, whereas in the E1KD cells, these transcripts were associated with the P fraction irrespective of TGFβ-treatment (Fig. 2B). This methodology accurately identified ILEI as one of the target transcripts, as demonstrated by semi-quantitative RT-PCR analysis of the sample cDNA used for the microarray hybridization (Fig. 2C). The ILEI mRNA is polyribosome-associated following TGFβ-treatment in parental NMuMG cells, whereas it is found polyribosome-associated in the E1KD cells in the absence or presence of TGFβ. Total ILEI mRNA levels were not affected by TGFβ stimulation in either cell type (Fig. 2C).

**Table 2:** List of 36 potential BAT genes identified by the combinatorial approach.

Accession	Gene Name	Fold Change Total mRNAin NMuMG	Fold Induction Polysomal mRNAin NMuMG	Fold ChangeTotal mRNAin E1KD	Fold Induction Polysomal mRNAin E1KD	Fold Change in hnRNP E1 binding
**BM232998**	2810474O19Rik	1.6934906	20.82147	1.574616	1.8150383	1.82134
**NM_015753**	Zeb2	1.8087588	21.33278	0.600818	1.5368752	2.386671
**U03425**	Egfr	1.0139595	5.856343	0.8066418	1.4240502	1.464086
**BC003232**	Actn1	1.5691682	6.254957	1.6529006	1.3013419	1.510473
**W30094**	0610010F05Rik	1.6021398	7.568461	1.2570134	1.0792282	1.389918
**AU067741**	D3Ertd254e	1.1850928	11.63178	1.0245568	1.6245048	1.552938
**NM_008413**	Jak2	1.7592982	6.727171	1.4339552	1.2141949	1.918528
**BB221842**	Sh3glb1	1.1526863	5.540438	1.2834259	1.3995859	1.82134
**AV357135**	Baz1a	1.0174797	14.02569	1.7411011	1.4948492	1.399586
**BE943736**	Asap1	1.1289644	5.063026	1.0717735	1.4896775	1.433955
**AK012196**	Pip4k2a	1.9520635	13.17746	1.201636	1.7171309	2
**BI662324**	Gna13	1.2184103	7.412704	0.9726549	1.866066	1.574616
**AV271901**	Eif5a2	1.1289644	6.988583	1	1.4590203	1.337928
**BC004850**	Twsg1	1.0867349	10.59271	0.9794203	1.4948492	1.274561
**BG071905**	Palld	1.6132835	9.57983	1.69937	1.5583292	1.735077
**X58380**	Hmga2	0.9106698	5.676493	1.8986842	1.3613141	1.274561
**AV174556**	Ubxn2a	1.2397077	7.621104	1.1566882	1.9453099	1.261377
**AK010212**	Pkia	1.7592982	13.04116	1.3472336	1.771535	1.36604
**BC025048**	Dusp7	1.270151	7.542276	1.5422108	1.5583292	2.136131
**BQ174163**	Tmem167	1.2483305	11.27457	1.2789856	1.6021398	1.310393
**NM_020296**	Rbms1	0.9233823	6.105037	0.8705506	1.7532114	1.30586
**AF065933**	Ccl2	1.0245568	5.521269	1.9185282	1.0069556	1.607702
**BF383782**	Tmem65	1.0245568	8.845845	1.082975	1.6934906	1.239708
**BC027138**	Zbtb44	1.2099941	5.169411	0.8150723	1.5052467	1.29684
**NM_008380**	Inhba	0.8321987	5.205367	1.1134216	1.5800826	1.274561
**NM_010833**	Msn	1.9453099	14.22148	1.3058598	1.9930805	1.892115
**BB148748**	Cast	1.6414832	11.47164	1.1809927	1.8150383	2.034959
**BM213828**	Kpna3	1.5691682	7.862565	1.3195079	1.8403753	1.494849
**AV127581**	2700049H19Rik	1.2483305	6.988583	1.5583292	1.1769067	1.618884
**BF119821**	Dpp8	1.1974787	8.907373	1.201636	1.7290745	1.252664
**NM_011544**	Tcf12	1.4590203	6.19026	1.082975	1.2397077	1.531558
**AW825881**	Zfp266	1.4142136	10.37472	1.6643975	1.0606877	1.515717
**AK017688**	5730469M10Rik	0.9896567	8.310873	1.053361	1.3707828	1.22264
**BB268102**	Phf20l1	0.952638	5.37029	1.531558	1.9793133	1.324089
**AF100171**	Mlf1	1.547565	17.44812	1.9318727	1.9656412	1.380317
**AK016470**	Fam3c	1.226885	7.260153	0.9362722	2.0849315	1.185093

Despite minor changes in total RNA levels, the target mRNAs display a >5 fold increase in polyribosome association in NMuMG cells post TGFβ treatment compared to E1KD cells where the target mRNAs display constitutive translational activation. Target mRNAs display a decrease in temporal association with hnRNP E1 following TGFβ stimulation for 24 hr.

Interestingly, several of the identified mRNAs have been previously implicated as targeted transcripts of TGFβ-mediated translational regulation including calpastatin [16] and epidermal growth factor receptor [17]. Additionally, this approach identified several candidates that have been shown to be involved in the EMT process including Eukaryotic initiation factor 5A2 [18], Moesin [19], Egfr [20] and Inhibin beta-A [21]. These candidates were subsequently used for further validation studies.

### Validation of Selected Genes from the Affymetrix Array

**Figure 3:**
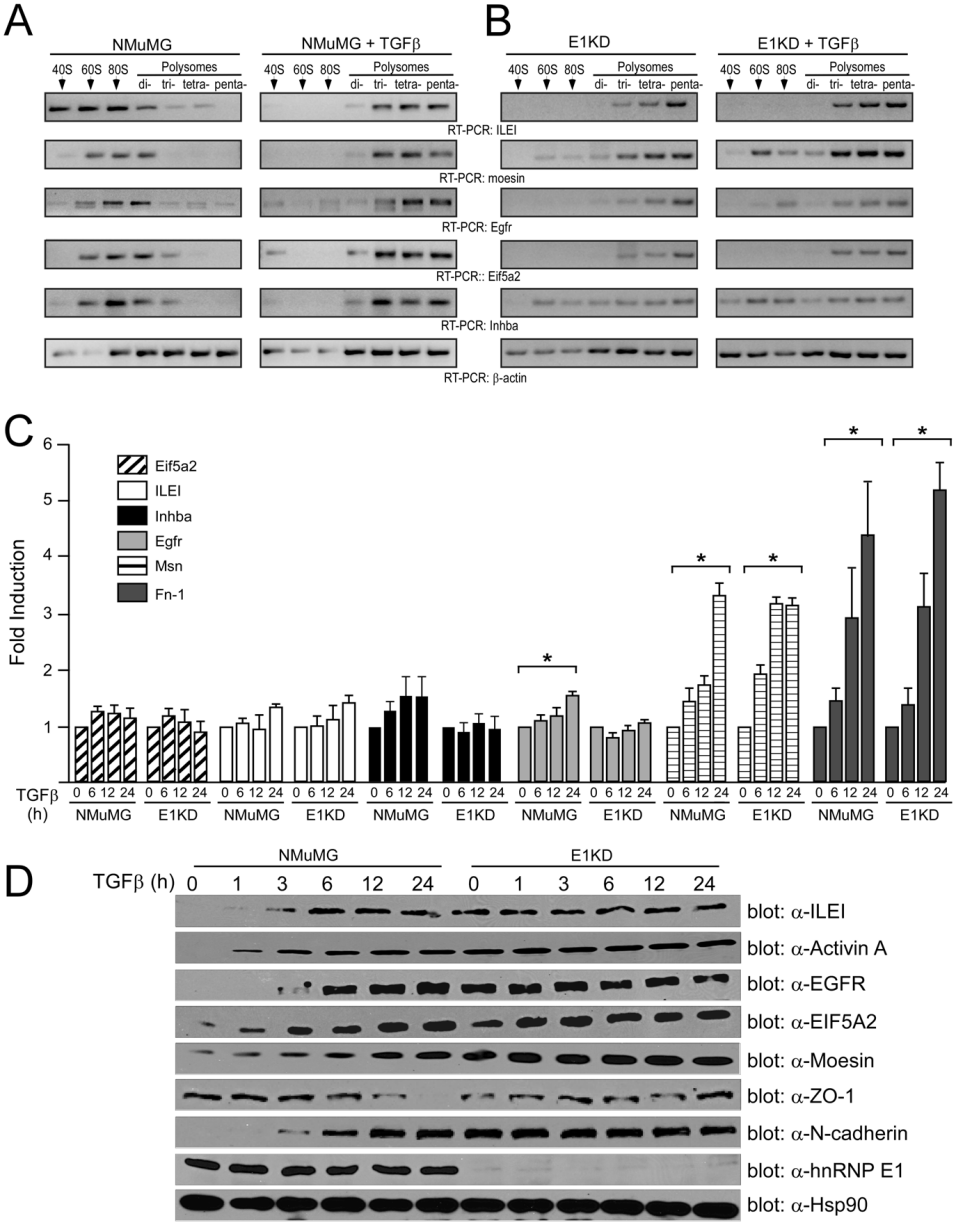
Select transcripts are used for further validation of the EMT signature gene targets. (A and B) RT-PCR analysis using gene specific primers for the potential targets and β-Actin (control) on a polysome profile of NMuMG and E1KD cells ± TGFβ for 24 hr. (C) Total RNA was isolated from NMuMG and E1KD cells treated with TGFβ for the indicated times and subjected to qPCR analysis to assess steady state mRNA expression levels. Data are presented as means ± s.e., n = 3 (*P<0.05). (D) Immunoblot analysis examining protein expression levels of the potential targets, α-Hsp90 (control) and α-N-cadherin and α-ZO-1 (EMT markers), in NMuMG and E1KD cells treated with TGFβ for the indicated times.

We next addressed whether the translational regulation of polysome-bound transcripts correlated with respective RNA and protein levels. Initially, we performed a polysome profile expression analysis independent of the pooled microarray samples to further demonstrate the translocation of mRNA from the non-translating M fractions to the actively translating P fractions in non-stimulated and TGFβ-treated cells. In parental NMuMG cells, the target mRNAs are primarily associated with the 80S fraction in non-stimulated cells, and display a complete shift to the actively translating polysomes after 24 h of TGFβ treatment (Fig. 3A). These results are in agreement with our previous findings that hnRNP E1-directed translational regulation targets the 80S stage of translation elongation [11]. In contrast, the hnRNP E1 knockdown E1KD cells displayed abundant target mRNA in the actively translating polysomal fractions irrespective of TGFβ treatment (Fig. 3B). As a control, semi-quantitative RT-PCR with β-actin specific primers displayed continuous association of the mRNA with the polysomes irrespective of TGFβ-treatment (Fig. 3A and Fig. 3B), demonstrating that the translational control is transcript-specific and not due to global regulation of translation.

TGFβ has been shown to exert both an antiproliferative effect, functioning as a tumor suppressor during early stages of tumorigenesis, whereas at later stages it functions as a tumor promoter aiding in metastatic progression through an autocrine TGFβ-loop [8]. For this reason we investigated whether the regulation of these BAT genes is specific to the induction of EMT by performing a polysome profiling analysis with a second *in vitro* model cell line, the well-polarized mammary epithelial cells (EpH4). EpH4 cells undergo cell cycle arrest and apoptosis in response to TGFβ-treatment, whereas the oncogenic Ha-Ras derivate (EpRas cells) will undergo EMT in response to TGFβ [10], [22], [23], [24]. The results from the polsome profile analysis reveal that the expression of these transcripts (total mRNA) did not vary significantly ± TGFβ in either the parental EpH4 or the derivative EpRas cells (Fig. S1A). However, in the EpRas cells, these transcripts preferentially translocated from the monosomal (M) to polysomal (P) fractions following TGFβ stimulation, whereas in the EpH4 cells, these transcripts were associated with the monosomal fraction irrespective of TGFβ-treatment (Fig. S1B), suggesting that TGFβ-mediated translational activation of these target mRNAs is specific to the EMT response.

We next investigated the temporal relationship between total mRNA levels and protein expression levels in TGFβ-treated NMuMG and E1KD cells. With the exception of moesin, total mRNA levels for these target genes, as measured by quantitative real time PCR (qPCR), displayed only minor changes following TGFβ-treatment in both the NMuMG and E1KD cells compared to a ∼5 fold increase in Fibronectin (Fn1), a mesenchymal marker and target of TGFβ-mediated transcriptional regulation (Fig. 3*C, D*). These results concur with the microarray data that demonstrate that total mRNA levels for these transcripts were only slightly induced by TGFβ (Fig. 2D). However, it cannot be completely excluded from these results that transcriptional regulation is involved, albeit at a low rate. In contrast, protein expression levels, as analyzed by immunoblot analysis (Fig. 3E), revealed that non-stimulated NMuMG cells, despite having abundant message, have low levels of protein for these target genes, and display a rapid, and time-dependent increase in protein expression levels following TGFβ-treatment (Fig. 3E). Furthermore, the increased protein expression levels of these transcripts were shown to correlate with acquisition of a mesenchymal phenotype as demonstrated by increased expression of the mesenchymal marker N-cadherin and decreased expression of Zona occludens 1 (ZO-1). In contrast, in the E1KD cells, although there is not an apparent reduction in the expression of epithelial cell marker ZO-1, the expression of the mesenchymal marker N-cadherin, as well as the protein expression levels of the BAT genes, were constitutive irrespective of TGFβ-treatment (Fig. 3E).

### Target mRNAs are Regulated through Interaction with hnRNP E1 and a Structurally Conserved BAT Element

**Figure 4:**
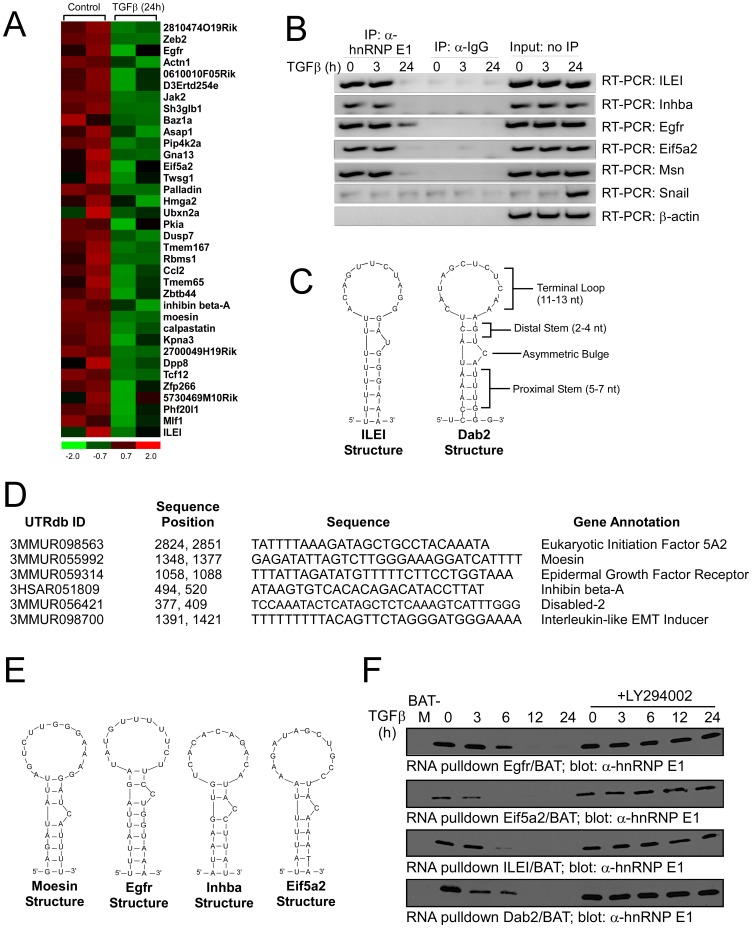
Identified mRNAs contain the BAT element and exhibit differential binding to hnRNP E1. (A) Heatmap of the RIP-Chip analysis for the putative EMT signature genes. (B) NMuMG cells were treated with TGFβ for times indicated, and RT-PCR was performed using gene specific primers for the potential targets, β-Actin (control) and Snail (EMT marker), on a RIP analysis. (C) Dab2/BAT and ILEI/BAT structures. Specific regions of the BAT element were selected and used to query the 3′-UTRs of the target mRNAs. (D and E) Secondary structures and sequences of target mRNAs with similarities to Dab2/BAT. (F) RNA affinity pull-down and immunoblot analyses to define the temporal association of hnRNP E1 with the selected BAT elements.

According to the RIP-Chip data, the selected target genes displayed a decrease in association with hnRNP E1 following TGFβ-treatment. The average signal intensity of the association with hnRNP E1 between control and TGFβ-treated samples are represented as a heat plot (Fig. 4A). In each case, less of these mRNAs were immunoprecipitated by α-hnRNP E1 in the presence of TGFβ compared to the control, unstimulated NMuMG cells (Fig. 4A). To further investigate the temporal association of hnRNP E1 with the selected target genes, we performed a RIP analysis independent of the microarray samples. As shown (Fig. 4B), hnRNP E1 interacts with the target transcripts. Immunoprecipitation with α-hnRNP E1 or mouse IgG from cytosolic extracts prepared from NMuMG cells treated with TGFβ for the times indicated, followed by RT-PCR analyses, revealed that while target mRNAs were steadily expressed, hnRNP E1 interaction occurred primarily in non-stimulated cells. These results are in agreement with our previous findings that TGFβ activates a kinase cascade terminating in the phosphorylation of hnRNP E1, by isoform-specific stimulation of protein kinase Bβ/Akt2, inducing the release of the hnRNP E1 from the 3′-UTR *cis* regulatory element, resulting in the reversal of translational silencing and increased expression of EMT-facilitating transcripts [10].

We have previously identified the structural BAT element in the 3′-UTRs of Dab2 and ILEI which binds hnRNP E1 and mediates TGFβ-induced translational regulation of these transcripts [10]. The Dab2 and ILEI BAT elements consist of a proximal stem and an asymmetric bulge followed by a distal stem and terminal loop (Fig. 4C). In order to determine whether the selected target genes also contain a respective BAT element, we utilized a consensus BAT element pattern, based on the secondary structure of Dab2 and ILEI BAT elements, to query the non-redundant 3′UTR sequences of the selected target genes using RNAmotif, an RNA secondary structure algorithm [25]. Putative BAT elements were identified in the target mRNAs with significant folding similarity as identified by the stem-loop and asymmetric bulge (Fig. 4D and Fig. 4E). Furthermore, we performed an additional *in silico* analysis of several of the target genes to determine if the predicted BAT elements are conserved in humans by querying the non-redundant 3′-UTR sequences of human ILEI, Eifa52 and Msn using the RNAmotif algorithm (Fig. S2). The results demonstrate that these target mRNAs share considerable sequence homology (>80%) as well as a conserved structural fidelity between the predicted mouse and human BAT elements (Fig. S2).

We next examined the temporal association of hnRNP E1 with the predicted BAT elements using an RNA affinity pull down assay (Fig. 4F). The respective BAT element cRNAs were coupled to sepharose beads, and used to precipitate hnRNP E1 from cytosolic S100 extracts isolated from TGFβ-treated and non-treated NMuMG cells. As a negative control, we used the Dab2/U10A element (BAT-M), which contains a U to A substitution at position 10 which unfolds the stem loop structure resulting in diminished binding affinity to hnRNP E1 [10],[11]. Immunoblot analysis confirmed that hnRNP E1 was precipitated by the predicted Egfr and Eif5a2 BAT elements from non-stimulated NMuMG, but TGFβ treatment induced the loss of hnRNP E1 binding in a time-dependent manner. Additionally, pre-treatment of NMuMG cells with the PI3K inhibitor LY294002, blocked the ability of TGFβ to modulate hnRNP E1 interactions (Fig. 4F), consistent with our previous observation that inhibition of the PI3K/Akt pathway blocked hnRNP E1 Ser43 phosphorylation [10].

## Discussion

Despite intensive transcriptional array analysis of human tumors, the identity and validation of ‘EMT signature genes’ remains elusive [26],[27],[28], partially because the transcriptome does not mirror the proteome [29]. To understand how the interplay of RNA-binding proteins affects the regulation of individual transcripts, high-resolution maps of *in vivo* protein-RNA interactions are necessary [30]. An alternative approach is expression profiling on a genome wide scale, whereby non-translating and actively translating pools of mRNAs are isolated by sucrose density gradient fractionation and subsequently subjected to microarray analysis [31]. RNA-Binding Protein Immunoprecipitation-Microarray (RIP-Chip) profiling is an advanced high-throughput analysis of mRNAs that co-immunoprecipitate with particular mRNA-binding proteins [32]. An mRNA-binding protein of interest is immunoprecipitated, and the associated mRNA is isolated and subsequently subjected to microarray analysis. A combinatorial approach involving expression profiling and RIP-chip analysis on a genome-wide basis will yield definitive information on a particular regulatory pathway.

Herein, we have identified a cohort of translationally regulated mRNAs that are upregulated during TGFβ-induced EMT by using a combinatorial approach involving polysome profiling and RIP-Chip analysis. Filtering the Affymetrix array data based on the translational state of transcripts in non-stimulated and TGFβ-treated NMuMG and E1KD cells, and intersecting these genes sets with the RIP-chip analysis led to the identification of a set of target mRNAs that follow the same pattern of regulation as Dab2 and ILEI, two transcripts necessary for EMT which were previously shown to be translationally regulated by TGFβ through hnRNP E1 [10],[11]. While our confidence in the establishment of this TGFβ-induced post-transcriptional EMT signature was strengthened by the identification of several transcripts which have been previously shown to be translationally regulated by TGFβ, including calpastatin [16] and epidermal growth factor receptor [17], our approach was not without some limitations. For example, this approach correctly identified ILEI mRNA, a well-characterized target for BAT-mediated translational silencing [10],[11], however, another previously identified target, Dab2 remained unidentified. This result may be due to a low signal-to-noise ratio of Dab2 expression levels, as the microarray based approach requires that the level of expression of target mRNA exceeds the cutoff limit of detection with a high-signal-to noise ratio [33].

Protein expression levels depend on the rate of transcription, as well as other defined control mechanisms, such as mRNA stability [34], nuclear export and mRNA localization [35], translational regulation [36], and protein degradation [37]. Post-transcriptional regulation is mainly controlled by the association of *trans*-acting RNA binding proteins with *cis*-regulatory regions in the UTRs of mRNAs. The bioinformatic prediction of putative BAT elements in the identified BAT mRNAs reveals a conserved structure-based homology based upon the functional structural motif previously identified for Dab2 and ILEI. These structures within the 3′-UTRs of the selected target mRNAs all share a stem-loop motif with an asymmetric bulge, albeit with considerable sequence diversity. Although the Egfr and Eif5a2 BAT elements were validated for their ability to bind hnRNP E1, a more comprehensive analysis is still required. This includes, but is not limited to fine mapping and cloning of the 3′-UTR of the candidate genes into reporter vectors to demonstrate functional gain-of-silencing potential. Furthermore, while *in silico* analysis demonstrated that the predicted BAT elements in several of the target mRNAs displayed considerable structural fidelity between mouse and human homologs, a more rigorous analysis is needed to determine if these conserved secondary structures are simply the result of of the high degree of intraspecies sequence homology in the respective 3′-UTRs, or the result of stabilizing selection.

The BAT element provides further insights into the importance of regulatory elements in the maintenance of homeostasis. Our results are suggestive of a stimulus-dependent upregulation of a post-transcriptional regulon coordinated by the concerted action of a *trans*-acting mRNP complex and a *cis*-regulatory element in the 3′-UTR of target genes. Eukaryotic regulons are defined as higher-order genetic units (quasi genome) consisting of monocistronic mRNA subsets under the control of a regulatory RNA binding protein [30]. RNA binding proteins have been shown to specifically bind transcripts encoding functional and colocalized protein classes [38],[39],[40]. Post-transcriptional regulons may have evolved as a mechanism to rapidly and coordinately suppress multiple EMT genes.

During the invasive phase of metastasis, a carcinoma cell activates EMT programs by different regulatory pathways. Differentiation to a mesenchymal phenotype enables the cancer cell with the ability to survive through the different steps of metastatic progression, including localized invasion by primary tumor cells, intravasation, translocation, extravasation and finally micrometastatic colonization at the secondary site [9]. Now, we demonstrate a cohort of selective transcripts that are post-transcriptionally upregulated by TGFβ and are correlative with an induction of the EMT phenotype. Akt2-mediated hnRNP E1 phosphorylation post-TGFβ stimulation is the regulatory mechanism mediating the TGFβ-induced translational activation of EMT-facilitating transcripts. We have now shown that hnRNP E1 is a central moiety in this process, and may represent an important molecular target for the development of modulators of this translational regulatory pathway. Furthermore, the continued delineation of the role of the identified target transcripts during EMT will prove to be extremely useful and will allow for their interrogation and manipulation in physiological and pathological situations.

## Methods

### Reagents

Mouse α-hnRNP E1 and α-ZO-1 were obtained from Novus Biologicals. α-ILEI, α-Inhibin beta A and α-EIF5A2 were obtained from Abcam. α-EGFR was obtained from Cell Signaling Technology. α-Moesin was purchased from BD Biosciences. α-Hsp90 and normal mouse IgG were purchased from Santa Cruz Biotechnology. Secondary antibodies, α-mouse and α-rabbit-IgG-HRP were obtained from GE Healthcare Bio-Sciences.

### Cell Culture and Treatments

Murine mammary epithelial NMuMG cells, obtained from American Type Culture Collection (ATCC; Manassas, VA, USA), were maintained in Dulbecco’s modified Eagle’s medium supplemented with 10% fetal bovine serum, 10 mg/ml insulin, and antibiotics/antimycotics (100 units/ml penicillin G, 100 mg/ml streptomycin, and 0.25 mg/ml amphotericin B). E1KD (previously termed SH14) were generated in the laboratory and have been described [10]. TGFβ2 was a generous gift from Genzyme Corporation (Cambridge, MA, USA) and was used at a final concentration of 5 ng/ml. Where indicated, cells were treated with 10 µM of LY294002 30 min before TGFβ treatment.

### Preparation of Cytosolic Extract (S100 Fraction)

S100 fractions were prepared from cells as previously described [41] with minor modifications. Briefly, the buffer used for cytosolic extraction contained 20 mM Hepes (pH 7.5), 10 mM KCl, 1.5 mM MgCl_2_, 1 mM EGTA, 1 mM EDTA, 1 mM DTT and protease inhibitor cocktail (Roche).

### RNA Immunoprecipitation

RNA immunoprecipitation was performed as described previously [32]. Briefly, the cytosolic extract was incubated with 10 µg of mouse α-hnRNP E1 antibody or mouse α-IgG at 4°C overnight, and precipitated with Protein A-Sepharose (Invitrogen). The beads were washed three times with IP Wash Solution (150 mM NaCl, 50 mM Tris pH 7.5, 0.5% NP40), and immunoprecipitated RNAs isolated by Trizol (Invitrogen) and treated with RNase-free DNase I (Applied Biosystems).

### Polysome Profiling

Polysome analysis was performed as described previously [42]. Briefly, cell lysates were layered onto a 10%-50% sucrose gradient and centrifuged at 100,000×g at 4°C for 4 h. Gradient fractions were collected using a fraction collector with continuous monitoring of absorbance at 254 nm. RNA was extracted with Trizol (Invitrogen) and purified with RNeasy minikit (Qiagen).

### Microarray Data Processing

Affymetrix microarray analysis was conducted on two independent samples for each experimental condition. Samples were processed at the MUSC Proteogenomics Facility (http://proteogenomics.musc.edu) using Affymetrix Mouse Genome 430 2.0 GeneChips® in accordance with the manufacturer protocols. The resulting raw data files were deposited in the NCBI Gene Expression Omnibus (Accession #GSE40466). Hybridization data (CEL files) were normalized by RMA algorithm using Affymetrix Expression Console software; detection calls were obtained by Affymetrix MAS5 algorithm. Gene representations not receiving ‘present’ detection scores in ≥25% of all samples were excluded from further analysis.

### Data Analysis

The average raw signal intensity values from two independent samples for each experimental condition were determined and used for multiparametric comparisons. Filtering of the genes sets met the following criteria: i) the ratio of the average raw signal intensity of monosomal (M) versus polysomal (P) mRNAs from control NMuMG cells was filtered as (M_control_/P_control_ ≥2), whereas in the E1KD cells the parameter was set at (P_control_/M_control_ ≥2); ii) the ratio of P vs. M associated mRNAs from TGFβ-treated NMuMG and E1KD cells was filtered as (P_TGFβ_/M_TGFβ_ ≥2); iii) fold change in total RNA from control vs. TGFβ-treated cells was determined by (Total RNA_TGFβ_/Total RNA_control_ ≤2); and, iv) the ratio of fold induction of polysomal RNA in NMuMG cells post TGFβ-stimulation compared to control was filtered at ([(P_TGFβ_/M_TGFβ_)/(P_control_/M_control_)] ≥5), whereas in the E1KD cells the parameter was set at ([(P_TGFβ_/M_TGFβ_)/(P_control_/M_control_) <2). Finally, for the RIP-Chip, the ratio of the average raw signal intensity for mRNAs immunoprecipitated by hnRNP E1 in control NMuMG cells vs. TGFβ-treated was filtered at (IP:E1 Control/TGFβ ≥1.2), whereas for the IgG immunoprecipitation the parameter was set at (IP:IgG Control/TGFβ ≤1).

### Functional Pathway Search Analysis

Functional analysis was performed using the Database for Annotation, Visualization and Integrated Discovery (DAVID), Molecular Signature Database (MSigDB) and Protein Analysis Through Evolutionary Relationships (Panther) platforms. Biological processes and pathway terms from Gene ontology (GO), Kyoto Encyclopedia of Genes and Genomes (KEGG), Panther, Reactome and Biocarta databases were utilized.

### Real-time Quantitative PCR (Taqman system)

Total RNA was isolated by Trizol extraction. Reverse transcription was performed using the Superscript first strand synthesis system (Invitrogen). Quantitative PCR was performed using an Applied Biosystems 7500 Fast Real-Time PCR System and default cycle conditions. Briefly, reactions were prepared using 50 ng cDNA, Taqman® Fast Universal PCR Master Mix and mouse-specific primers for Egfr (cat number Mm00433023_m1), Moesin (Mm00447889_m1), Eif5a2 (Mm00812570_g1), Fam3c (Mm00506842_m1), Inhba (Mm00434339_m1), Fn1 (Mm01256744_m1), and Gapdh (Mm99999915_g1) according to manufacturer’s protocol (Life technologies). All samples were run in triplicate and normalized to Gapdh. Data analysis was performed using the relative quantification (ΔΔC_T_) method [43].

### RNA Pulldown

RiboMax kit (Promega) was used to generate milligram quantity of BAT cRNA. cRNA was bound to CNBr-activated Sepharose beads and incubated for 1 h at 4°C with 50 µg of cytosolic extract (S100 Fraction) from NMuMG cells treated ± TGFβ. Following incubation, beads were washed with 0.2 M NaCl and resolved by SDS-PAGE.

### Bioinformatic Prediction of BAT Elements

Analysis of select 3′UTR genes was completed using RNAmotif software [25] on a MacBook Pro using an Intel Core-i7, 4 gb of RAM, and Mac OS-X 10.6. Software was compiled using GNU’s GCC compiler (gcc.gnu.org) in the OS-X terminal utility. Descriptor file was written using Xcode version 3.2 in the C development module. Input file was created in a generic text editor, sequences were obtained from UTRdb (utrdb.ba.itb.cnr.it). Analysis was run from the OS-X terminal utility, output was sent to a generic text file to be used for later interpretation.

## Supporting Information

Figure S1 **Validation of the putative EMT signature gene targets in EpH4 and EpRas cells (related to** **Figure 3** **).** (A) RT-PCR analysis using gene specific primers for the potential targets and β-Actin (control) on total RNA extracted from EpH4 and EpRas cells ± TGFβ for 24 hr. (B) Monosomal fractions (M; 40S, 60S, and 80S fractions) and polysomal fractions (P) from EpH4 or EpRas cells treated ± TGFβ for 24 hr were isolated by sucrose gradient centrifugation and pooled. RT-PCR analysis using gene specific primers for the potential targets and β-Actin (control).
(DOC)

Figure S2 ***In silico*** **analysis of BAT elements from human homologs of target mRNAs (related to** **Figure 4** **).** (A, B, C) Comparison of secondary structures and sequences of BAT elements from human and mouse target BAT genes. Specific regions of the BAT element were selected and used to query the human 3′-UTRs of (A) ILEI/BAT, (B) Moesin/BAT structures, and (C) Eif5a2/BAT constructs. Sequence homology is indicated by starred (*) nucleotides.
(DOC)
