# Supplementary figures and images for: Establishment of a TGFβ-Induced Post-Transcriptional EMT Gene Signature
Source: PLoS One. 2012 Dec 20;7(12):e52624. doi: 10.1371/journal.pone.0052624 (PMC3527574; doi:10.1371/journal.pone.0052624)

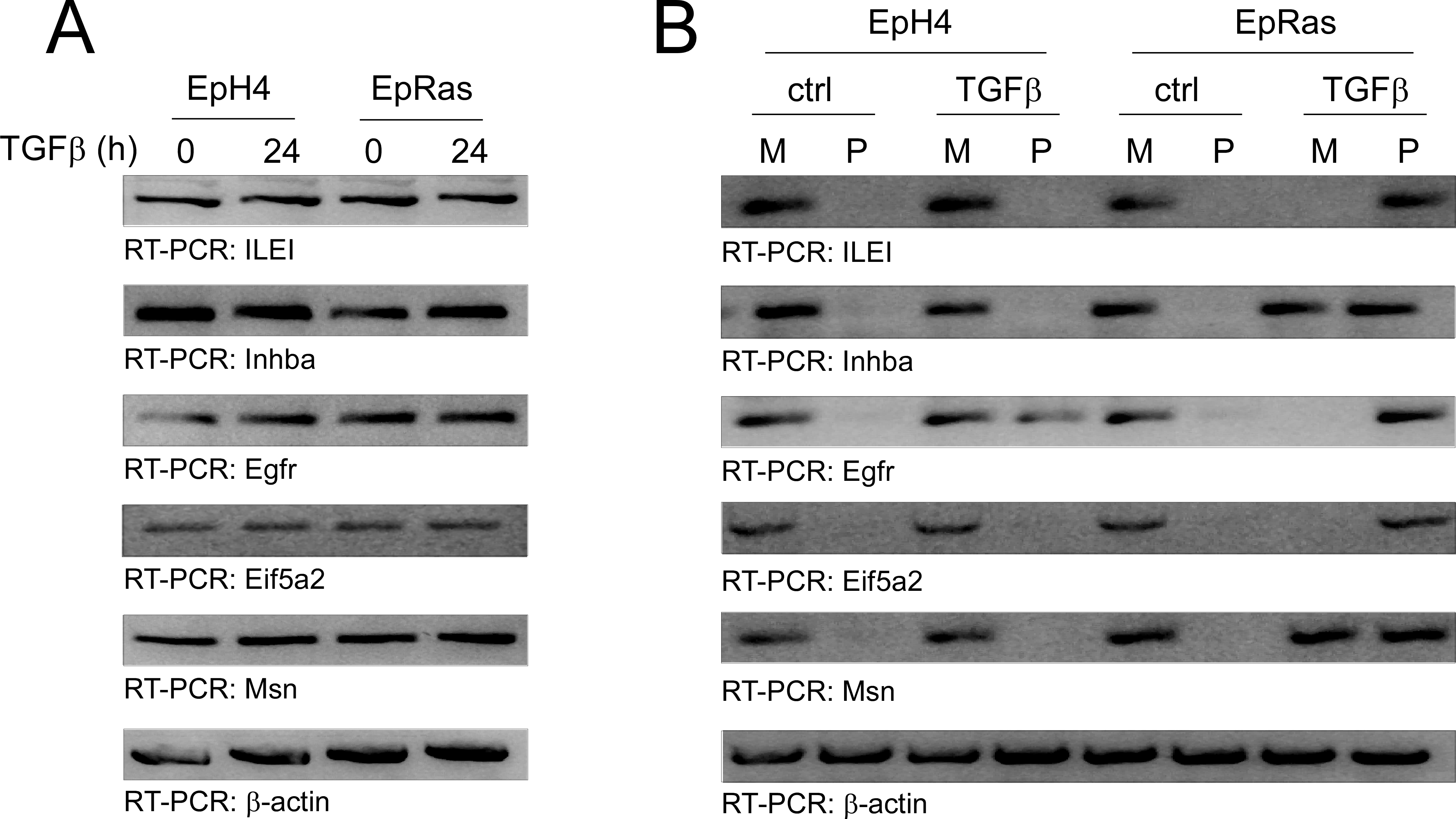

Supplement: Figure S1 — Validation of the putative EMT signature gene targets in EpH4 and EpRas cells (related to Figure 3 ). (A) RT-PCR analysis using gene specific primers for the potential targets and β-Actin (control) on total RNA extracted from EpH4 and EpRas cells ± TGFβ for 24 hr. (B) Monosomal fractions (M; 40S, 60S, and 80S fractions) and polysomal fractions (P) from EpH4 or EpRas cells treated ± TGFβ for 24 hr were isolated by sucrose gradient centrifugation and pooled. RT-PCR analysis using gene specific primers for the potential targets and β-Actin (control). (DOC) [file pone.0052624.s001.doc]

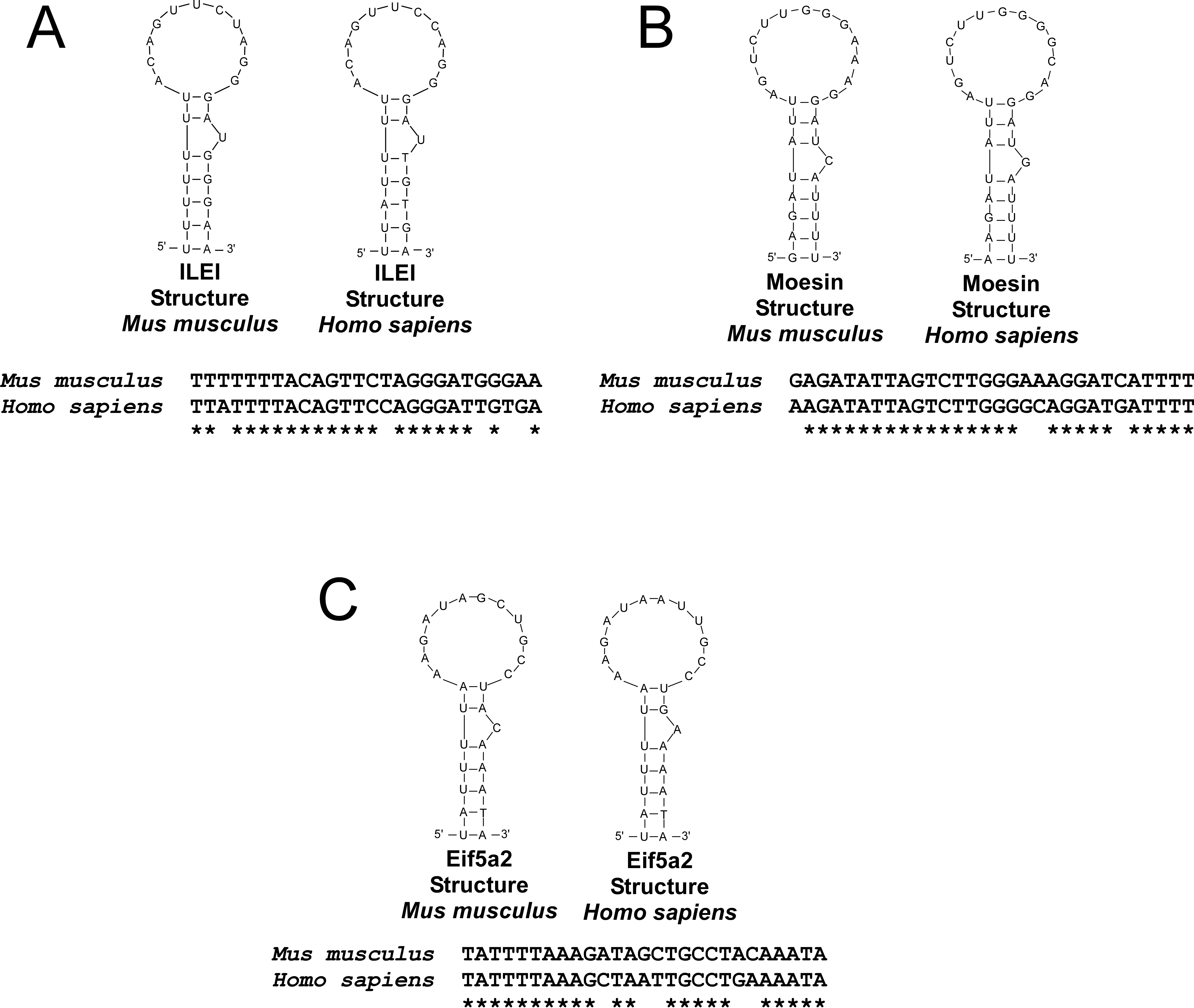

Supplement: Figure S2 — In silico analysis of BAT elements from human homologs of target mRNAs (related to Figure 4 ). (A, B, C) Comparison of secondary structures and sequences of BAT elements from human and mouse target BAT genes. Specific regions of the BAT element were selected and used to query the human 3′-UTRs of (A) ILEI/BAT, (B) Moesin/BAT structures, and (C) Eif5a2/BAT constructs. Sequence homology is indicated by starred (*) nucleotides. (DOC) [file pone.0052624.s002.doc]
